# Different Effects of Supervisor Positive and Negative Feedback on Subordinate In-Role and Extra-Role Performance: The Moderating Role of Regulatory Focus

**DOI:** 10.3389/fpsyg.2021.757687

**Published:** 2022-01-07

**Authors:** Weilin Su, Shuai Yuan, Qian Qi

**Affiliations:** ^1^School of Literature, Capital Normal University, Beijing, China; ^2^School of Management Engineering and Business, Hebei University of Engineering, Handan, China; ^3^School of Artificial Intelligence, Beijing University of Posts and Telecommunications, Beijing, China

**Keywords:** supervisor positive feedback, supervisor negative feedback, in-role performance, extra-role performance, regulatory focus

## Abstract

As an important tool for supervisors to intervene subordinates’ work and influence their performance, supervisor feedback has gradually become a new academic research hotspot. In this study, we build and verify a theoretical model to explore the different effects of supervisor positive and negative feedback on subordinate in-role and extra-role performance, and the moderating role of regulatory focus in these relationships. With data from pairing samples of 403 Chinese employees and their direct supervisors, the results indicate that supervisor positive feedback is positively related to subordinate in-role and extra-role performance. Supervisor negative feedback is positively related to subordinate in-role performance and negatively related to subordinate extra-role performance. Regulatory focus of subordinate can moderate the influence of supervisor positive feedback on subordinate in-role and extra-role performance, but it cannot moderate the influence of supervisor negative feedback on subordinate in-role and extra-role performance. That means when subordinates have promotion focus, the influence of supervisor positive feedback on their in-role performance and extra-role performance was stronger than those with prevention focus. These results further enrich the research on the relationship between supervisor feedback and subordinate performance, especially the different effects of positive and negative feedback from supervisor on subordinate with different regulatory focus. All conclusions from the analyses above not only further verify and develop some previous points on supervisor feedback and subordinate performance, but also derive certain management implications for promoting subordinate in-role and extra-role performance from the perspective of supervisor positive and negative feedback.

## Introduction

How to improve organizational performance has been the focus of many scholars (e.g., Burke and Litwin, 1992; Richard et al., 2009; Fahrenkopf et al., 2020; Li C. et al., 2020), and the key to the level of organizational performance depends on individual performance (Katz and Kahn, 1978; Janardhanan et al., 2020). Individual performance can be further divided into in-role performance (Williams and Anderson, 1991; Wingerden and Poell, 2017) and extra-role performance (Becker and Kernan, 2003; Eisenberger et al., 2010). In-role performance refers to the quantity and quality of job performance that is defined in the organization and should be achieved within the scope of responsibilities (Katz and Kahn, 1978), which can correspond to individual in-role behavior and task performance to a certain extent. Extra-role performance refers to individual’s spontaneous activities in the organization that exceed his/her job role and the requirements of the organizational reward system (Becker and Kernan, 2003), which can be corresponding to the individual extra-role behavior (Van Dyne et al., 1994) and situational (non-task) performance.

A large number of studies have been conducted on the factors influencing individual in-role and extra-role performance. For example, from the perspective of individual, numerous scholars have discussed the influence of personality traits, ability, motivation, values and skills on individual performance (e.g., Barrick and Mount, 1991; Tett and Burnett, 2003; Rich et al., 2010). And other scholars have studied the effect of leadership on subordinate performance, such as leadership behavior, leadership style, leadership intervention and so on (Ashford and Northcraft, 1992; Judge et al., 2006; Wong and Laschinger, 2013). Supervisor feedback, as an important tool for leaders to intervene in subordinates’ daily work (Su et al., 2019), is likely to have a significant impact on subordinate in role and extra-role performance. Supervisor feedback can be divided into positive and negative (Jaworski and Kohli, 1991; Zheng et al., 2015). Positive feedback emphasizes that supervisors treat their subordinates in an affirmative, inclusive and encouraging way, while negative feedback emphasizes that supervisors treat their subordinates in a critical, frustrating and disapproving way.

Generally speaking, subordinates are more willing to accept positive feedback from supervisors than negative feedback (Audia and Locke, 2003; Layous et al., 2017). In other words, supervisor positive feedback is more likely to lead to positive responses from subordinates. However, in terms of the effect of supervisor feedback on subordinate performance, a meta-analysis by Kluger and DeNisi (1996) found that 62% of studies confirmed that feedback had a positive effect on performance and 38% confirmed that feedback had a negative effect on performance. Some scholars have also pointed out that supervisor negative feedback does not necessarily harm the organization, and in some cases, negative feedback is even more valuable than positive feedback (Trope and Neter, 1994). That is to say, the process mechanism and boundary conditions of the influence of supervisor positive and negative feedback on subordinate performance have not been well analyzed by existing studies, and the influences of the two forms of supervisor feedback on subordinates are quite different. Therefore, it is necessary to subdivide supervisor feedback and subordinate performance, and further explore the different effects of supervisor positive and negative feedback on subordinates in-role and extra-role performance, respectively.

Regulatory Fit Theory emphasizes that when the external situational factors are matched with the individual regulatory focus, individuals can be promoted to have more feelings of rightness and emotional experience of importance for their own behaviors (Crowe and Higgins, 1997; Simmons et al., 2016). This can also bring more “Value from Fit” (Higgins, 2005), which can improve individual work motivation, attitude, behavior and performance. And, Higgins (2000) pointed out that individuals can self-regulate their own cognition and behavior in the process of goal realization, and may adopt two kinds of regulatory strategies (promotion and prevention) based on satisfying their own different demands, which will have an impact on their attitude, behavior and even performance. Therefore, regulatory focus of subordinate is an important individual factor that affects the relationship between supervisor and subordinate (Shin et al., 2017), and plays an important role in predicting their own work behavior and performance (Wallace et al., 2009). In view of the different effects of individual regulatory focus, whether the different effects of supervisor positive and negative feedback on subordinate in-role performance and extra-role performance are affected by them? This is also a topic worth discussing. Hence, this study also introduced regulatory focus of subordinate into the model as a moderating variable to further explore the boundary condition of the influence effect between supervisor feedback and subordinate performance.

This study contributes to the literature in the following three ways. First, we attempt to discuss the different effects of supervisor positive feedback and negative feedback on subordinate in-role and extra-role performance. This not only discusses the influencing factors of subordinate performance from the perspective of supervisor feedback (Kluger and DeNisi, 1996; Kuvaas et al., 2017; Su et al., 2019), but also can more clearly and comprehensively analyze the specific influences of different types of supervisor feedback on subordinate performance. Second, we introduce chronic regulatory focus of subordinate as moderator into these relationships to identify the unique boundary conditions for supervisor positive feedback and negative feedback to affect subordinate in-role and extra-role performance. This can deepen the understanding of the connotation, applicability and explanatory power of regulatory focus theory (Higgins, 1997, 2000; Lockwood et al., 2002; Vaughn, 2017). Third, since supervisor feedback has an important impact on subordinates’ work attitude, behavior and performance (Zhang et al., 2017; Eva et al., 2019), this study can provide management enlightenment for organizations and their managers on how to implement effective feedback management strategies to maximize subordinate performance.

**FIGURE 1:**
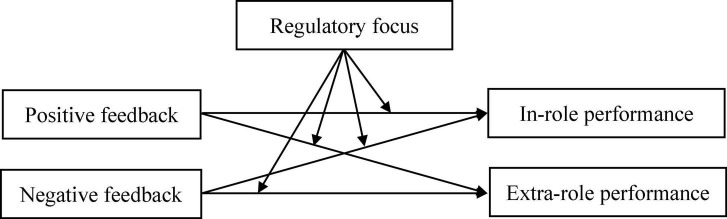
Theoretical model.

In summary, this study aims to build a theoreticall model to explore the different effects of supervisor positive feedback and negative feedback on subordinate in-role performance and extra-role performance, and discuss the moderating role of regulatory focus in these relationships. The overall theoretical model of this study is presented in Figure 1.

## Theoretical Background

### Supervisor Positive Feedback and Subordinate Performance

Supervisor positive feedback means that the supervisor treats subordinates in a positive way and emphasizes the positive evaluation of subordinates’ working attitude, behavior and results. It is a form of reinforcing feedback that is perceived by subordinates as recognition, encouragement or support from their supervisors (Zheng et al., 2015). In terms of the influence of supervisor positive feedback on subordinates, many existing studies have drawn a relatively consistent conclusion that supervisor positive feedback has a relatively positive impact on work attitude, behavior and performance of subordinates (e.g., Zhou, 2003; Steelman et al., 2004; Peng and Chiu, 2010; Su et al., 2019). The following will elaborate the internal logic of the influence of supervisor positive feedback on subordinates in-role and extra-role performance from the perspective of in-role and extra-role.

In-role performance refers to core task behaviors that directly or indirectly contribute to individual and organizational productivity (Katz and Kahn, 1978). It is closely related to individual work experience, ability, skill and knowledge (Borman and Motowidlo, 1997). For the influence of supervisor positive feedback on subordinate in-role performance, positive feedback emphasizes the positive evaluation on the subordinates’ working attitude and behavior, which can maintain and improve the self-esteem and confidence of subordinates and make them feel that they can get support and trust from leaders and organizations (Dahling et al., 2017). This also further improves subordinates’ ability to think and solve work problems, thus showing a higher level of in-role performance. On the flip side, supervisors can create a relaxed and free atmosphere in the organization through positive feedback (Dahling and O’Malley, 2011). Such an atmosphere can bring subordinates a higher level of satisfaction and happiness, and make them more willing to actively complete the tasks prescribed by the organization, which is also more conducive to the improvement of subordinate in-role performance (Ghosh et al., 2017). Therefore, we infer that supervisor positive feedback may have a positive impact on subordinate in-role performance.

In terms of the effect of supervisor positive feedback on extra-role performance, positive feedback can effectively improve subordinate’ competences and autonomy at work, and then enhance their intrinsic motivation (Ilgen and Davis, 2000; Su et al., 2019). And intrinsic motivation is the key factor for individuals to show the extra-role behaviors that are not mandatory by the organization (Tu and Lu, 2016). Therefore, supervisor positive feedback can promote intrinsic motivation of subordinates, so that subordinates can produce extra-role performance. Besides, supervisors can transmit positive support signals to subordinates through positive feedback (London, 1995), which also effectively improves the quality of the relationship between supervisors and subordinates. And the quality of this exchange relationship is the key factor affecting the subordinate extra-role performance (Cropanzano and Mitchell, 2005). Hence, we believe that supervisor positive feedback also has a positive impact on subordinate extra-role performance. In conclusion, the following hypothesis is proposed:

*Hypothesis 1a: Supervisor positive feedback is positively related to subordinate in-role performance.*
*Hypothesis 1b: Supervisor positive feedback is positively related to subordinate extra-role performance.*

### Supervisor Negative Feedback and Subordinate Performance

Supervisor negative feedback refers to the supervisors treating their subordinates in a negative way and emphasizing the correction of subordinates’ bad or ineffective work behaviors. It is a form of corrective feedback that is negative, critical, or unsupportive from their supervisors (Herold et al., 1987; Zheng et al., 2015). As for the influence of supervisor negative feedback on subordinate performance, the academic circle has not formed a unified cognition. For example, Podsakoff and Farh (1989) confirmed that supervisor negative feedback had a more obvious role in promoting subordinate performance, while Dahling et al. (2017) found that supervisor negative feedback could hardly promote subordinate performance. The reason for this inconsistency may be that the two-dimensional concept of performance is only discussed as a whole and cannot accurately reflect the differences and connections between in-role performance and extra-role performance. Therefore, it is difficult to clarify the different effects of supervisor negative feedback on subordinate in-role performance and extra-role performance.

In terms of the influence on subordinate in-role performance, this study believes that supervisor negative feedback can also promote subordinate in-role performance. On the one hand, negative feedback from supervisors, especially from trustworthy supervisors, can make subordinates fully recognize their possible deficiencies, help them set more scientific and reasonable goals (Fedor et al., 2001), and constantly adjust their work behaviors according to these goals, so as to achieve the purpose of improving subordinate in-role performance (Podsakoff and Farh, 1989; Audia and Locke, 2003). On the other hand, it is inevitable that subordinates may have bad or ineffective work behaviors in their daily work. When supervisors give clear corrections through negative feedback, subordinates can timely realize their own biases, problems and mistakes (Waldersee and Luthans, 1994; Ilgen and Davis, 2000) to help subordinates to better complete work tasks (Henley and DiGennaro Reed, 2015), which can also promote subordinates to show higher in-role performance. Therefore, we believe that supervisor negative feedback has a positive impact on subordinate in-role performance.

As mentioned above, negative feedback is when supervisors treat their subordinates in a critical, frustrating and disapproving way (Herold et al., 1987), which indicates that the daily work of subordinate does not meet the standards expected by the organization. For subordinates, this means that some of their work tasks have failed, which hurts the self-esteem of subordinates and leads to negative and pessimistic emotional experience (Spector and Fox, 2002). Such emotional experience is likely to make subordinates fall into a negative working attitude and even unwilling to accept negative feedback (Kim and Kim, 2020), so they are unwilling to make corresponding behavior improvement on the basis of negative feedback (Fedor et al., 1989). Not to mention actively participating in extra-role activities that are not mandated by the organization. In addition, supervisor negative feedback is often inconsistent with subordinates’ own positive cognitive expectations, which leads the subordinate to think that their failure is inevitable and it is difficult to achieve the expected goals no matter how hard they try (Abramson et al., 1978; Chughtai et al., 2015). Once employees have such an idea, they will usually actively avoid all activities with risks (Lemoine et al., 2019), and it is difficult for them to engage in the extra-role activities that may be risky for themselves but beneficial to the organization, resulting in a low level of extra-role performance. Therefore, we believe that supervisor negative feedback has a negative impact on subordinate extra-role performance. In conclusion, the following hypothesis is proposed:

*Hypothesis 2a: Supervisor negative feedback is positively related to subordinate in-role performance.*
*Hypothesis 2b: Supervisor negative feedback is negatively related to subordinate extra-role performance.*

### The Moderating Role of Regulatory Focus

Regulatory focus, as a relatively stable individual characteristic, is formed in the process by which individual seek to align with appropriate goals or standards (Higgins, 2000). It is closely related to the individual’s specific motivation, behavioral strategy and information processing mode (Werth and Foerster, 2007), and can be further divided into two specific types: promotion focus and prevention focus (Higgins, 1997, 2000). Generally speaking, individuals with promotion focus are more sensitive to the positive outcomes and are more focused on vision, expectation, and gains. It reflects the individuals’ needs to pursue “ideal self,” and pays more attention to what can be brought to the individuals after the successful realization of the goals. Individuals operating primarily with prevention focus are more concerned about the negative outcomes, and more focused on duty, responsibility, and losses. It reveals the individuals’ pursuit of “moral self” and focuses more on what the individual will lose if the goal is not achieved (Higgins, 1997; Brockner and Higgins, 2001; Li C. et al., 2020).

In this study, regulatory focus of subordinate is expected to play a moderating role in the relationship between supervisor feedback (incl. positive feedback and negative feedback) and their performance (incl. in-role performance and extra-role performance). Subordinates with promotion focus are more sensitive to the presence or absence of positive things, which means that subordinates with promotion focus are more likely to respond when supervisors provide positive feedback (Righetti and Kumashiro, 2012). They are more likely to adjust their work attitudes and behaviors based on the positive feedback from their supervisors, thus showing a higher level of performance. Besides, compared to individual with prevention focus, promotion-focused subordinates have higher internal acceptance of positive feedback from supervisors. Naturally, they are more willing to use the information delivered by their supervisors through positive feedback to improve their own in-role and extra-role performance. Therefore, the following hypothesis is proposed:

*Hypothesis 3a: Regulatory focus moderates the relationship between supervisor positive feedback and subordinate in-role performance, such that this relationship would become stronger for subordinates with promotion focus.*
*Hypothesis 3b: Regulatory focus moderates the relationship between supervisor positive feedback and subordinate extra-role performance, such that this relationship would become stronger for subordinates with promotion focus.*

Compared with prevention-focused individuals, the employees with promotion focus pay less attention to negative events in the organization (Lockwood et al., 2002), and therefore when supervisors provide feedback to subordinates in a negative way, promotion-focused subordinates are less affected than those with prevention focus. Meanwhile, when subordinates with prevention focus perceive negative feedback from supervisors, they tend to adopt defensive goal realization strategy (Brockner and Higgins, 2001) and they are more negative instead of taking various solutions to cope with such supervisor feedback (Lanaj et al., 2012), so it is difficult to obtain the change of their in-role and extra-role performance. In addition, subordinates with promotion focus are more optimistic and positive when they are faced with risks, pressures and setbacks (Hamstra et al., 2011). In other words, they can respond to the negative feedback from supervisors in a more positive and optimistic state, which also alleviates the damage caused by negative feedback to subordinates (Steelman and Rutkowski, 2004; Whitman et al., 2014). To sum up, subordinates with prevention focus are more sensitive to supervisor negative feedback than those with promotion focus. Therefore, we assume that regulatory focus of subordinates can negatively regulate the relationship between the supervisor negative feedback and their performance, and propose the following hypothesis:

*Hypothesis 3c: Regulatory focus moderates the relationship between supervisor negative feedback and subordinate in-role performance, such that this relationship would become stronger for subordinates with promotion focus.*
*Hypothesis 3d: Regulatory focus moderates the relationship between supervisor negative feedback and subordinate extra-role performance, such that this relationship would become stronger for subordinates with promotion focus.*

## Materials and Methods

### Samples and Procedures

The employees and their immediate supervisors who provide valid responses for two waves of this study from two industrial companies and one financial company in Chongqing and Sichuan Province in China. The authors contacted the HRDs of these three companies through personal networks and asked them to assist in the investigation. We also selected and trained a key contact person from each company to distribute and collect questionnaires, further improving the scientific nature of data collection.

In order to reduce the Common Method Variance, this study adopted Podsakoff et al.’s (2012) suggestions and got the data at two different times from two-source. At Time 1, 600 employees were invited to fill in the questionnaire, including their perceptions of supervisor positive feedback, negative feedback, regulatory focus and demographic information (e.g., gender, age, education level, and work tenure with their direct supervisor). One month later (Time 2), we asked the immediate supervisors of the 600 employees who took part in the first survey to evaluate their subordinates’ in-role and extra-role performance.

After excluding those invalid questionnaires (e.g., the questionnaire could not be matched, the options showed obvious regularity, or the entire questionnaire had the same option), the final sample of this study included 403 employees and their supervisors, representing a response rate of 67.17%. Demographic information indicated that 54.1% were male, and 45.9% were female. 68.5% of the participants hold a bachelor’s degree or above. In terms of age, the subordinates involved in this study were mainly young people, of which 62.3% are between 23 and 30 years old. The average work tenure with their direct supervisors were 25.13 months.

### Measures

In this study, the original scales for all core variables were built in English, so they had to be translated into Chinese first. We followed the translation and back-translation produces (Brislin, 1983) to create the Chinese versions of core scales to measure supervisor positive feedback, negative feedback, subordinate in-role performance, extra-role performance and regulatory focus. All items were rated on a 5-point Likert scale (1 = Strongly disagree and 5 = Strongly agree).

### Supervisor Positive and Negative Feedback

This study utilized the supervisor feedback scale designed by Jaworski and Kohli’s (1991). This scale consists of eighteen items and two subscales. Positive feedback subscale has nine items, and an example item is “I often receive positive feedback from my supervisor.” The Cronbach’s a of this subscale in this study was 0.809. Negative feedback subscale also has nine items, and an example item is “When I make mistakes at work, my supervisor usually tells me directly.” The Cronbach’s a of this subscale in this study was 0.935.

### Subordinate In-Role Performance

To measure subordinate in-role performance, this study adopted Williams and Anderson’s (1991) seven-item scale. The scale focuses on evaluating the tasks that subordinates need to complete in their daily work, and invites direct supervisors to evaluate job performance of subordinates. An example item is “This subordinate adequately completes assigned duties by me.” The Cronbach’s a of this subscale in this study was 0.917.

### Subordinate Extra-Role Performance

To measure subordinate extra-role performance, this study used the eight-item scale developed by Eisenberger et al. (2010). This scale invites the supervisors of the organization to evaluate the extra-role performance of their subordinates from the aspects of constructive suggestions, improving their own knowledge and skills, protecting the organization and helping colleagues. An example item is “This subordinate is often actively looking for new ways to improve productivity at work.” The Cronbach’s a of this subscale in this study was 0.926.

### Regulatory Focus

The chronic regulatory focus scale designed by Lockwood et al. (2002) was adapted in this study. This scale focuses on the realization degree of individual goal state, and is the most widely used scale to measure chronic regulatory focus (Gorman et al., 2012). This scale consists of eighteen items and two subscales. Promotion focus has nine items, and an example item is “I usually focus on things that might be successful in the future.” Prevention focus also has nine items, and an example item is “I always think about how to avoid failure.” The level of subordinates’ dominant regulatory focus by subtracting scores on the prevention regulatory subscale from scores on the promotion regulatory subscale (Lockwood et al., 2002). Higher scores on this measure reflect relatively greater promotion than prevention focus. The whole Cronbach’s a of this scale in this study was 0.879.

### Control Variables

Relevant studies (e.g., Zhou, 2003; Gabriel et al., 2014; Su et al., 2019) have shown that demographic characteristics such as gender, age, education and working tenure of subordinates can affect their response to supervisor feedback. At the same time, some scholars pointed out that individual characteristics such as age and gender would also have an impact on their performance (Thompson, 2005; Li X. et al., 2020). Hence, the gender, age, education and tenure were taken as control variables in the analyses to more accurately grasp the different effects of supervisor positive and negative feedback on subordinate in-role and extra-role performance.

### Analytic Strategy

This study used SPSS 22.0 and Mplus 7.0 software to analyze the final sample data. At first, we employed the confirmatory factor analysis (CFA) to assess the convergent and discriminant validity of the key variables using Mplus 7.0 (Anderson and Gerbing, 1988). Then, we performed descriptive analyses and correlation analysis to describe the participants’ demographic characteristics and initially test the relationship between the variables using SPSS 22.0. Finally, we adopted the multiple linear regression to test the hypotheses, and the bootstrap analysis to further verify the moderating role of regulatory focus using the SPSS macro-PROCESS (Cohen et al., 2003; Preacher et al., 2010; Hayes, 2015).

## Results

### Confirmatory Factor Analysis

The results of CFAs are shown in Table 1. It indicates that the hypothesized five-item model (i.e., supervisor positive feedback, negative feedback, regulatory focus, in-role performance and extra-role performance) fits the data better than other nested models (χ^2^/*df* = 1.633, RMSEA = 0.048, CFI = 0.916, TLI = 0.906, SRMR = 0.045). Therefore, we conclude that the measures of five core variables in this study can clearly distinguish the constructs.

**TABLE 1:** Results of CFAs: comparison of measurement models.

Models	Factors	χ^2^/*df*	RMSEA	CFI	TLI	SRMR
Model 1	Five factors: SPF, SNF, RF, IRF, ERP	1.633	0.048	0.916	0.906	0.045
Model 2	Fours factors: SPF + SNF, RF, IRF, ERP	1.956	0.057	0.836	0.824	0.059
Model 3	Fours factors: SPF, SNF, RF, IRF + ERP	1.844	0.055	0.859	0.847	0.047
Model 4	Three factors: SPF + SNF, RF, IRF + ERP	2.662	0.078	0.637	0.622	0.084
Model 5	Two factors: SPF + SNF, RF, IRF + ERP	3.131	0.089	0.510	0.492	0.102
Model 6	One factors SPF + SNF + RF + IRF + ERP	5.528	0.135	0.266	0.236	0.157

*N = 403.*

*SPF represents supervisor positive feedback; SNF represents supervisor negative feedback; RF represents regulatory focus; IRP represents in-role performance; ERP represents extra-role performance; Deal model-fit indicators are: χ^2^/df < 3, RMSEA < 0.08, CFI > 0.9, TLI > 0.9, SRMR < 0.08.*

### Descriptive Statistical Analysis

The results of descriptive analyses and correlation analysis are presented in Table 2. It reveals that supervisor positive feedback is positively related to subordinate in-role performance (*r* = 0.298, *p* < 0.01) and subordinate extra-role performance (*r* = 0.221, *p* < 0.01). Supervisor negative feedback is positively related to subordinate in-role performance (*r* = 0.352, *p* < 0.01), and negatively related to subordinate extra-role performance (*r* = −0.174, *p* < 0.01). Taken together, these are consistent with the hypotheses proposed in this study.

**TABLE 2:** Means, standard deviations, and bivariate correlations among studied variables.

Variables	1	2	3	4	5	6	7	8	9
1. Gender	1								
2. Age	0.080	1							
3. Education	0.091	0.248**	1						
4. Tenure	–0.057	–0.019	0.286**	1					
5. Positive feedback	–0.039	−0.136**	–0.025	0.026	1				
6. Negative feedback	0.018	–0.074	–0.033	–0.008	–0.093	1			
7. Regulatory focus	0.001	0.009	–0.008	0.061	0.145**	0.171**	1		
8. In-role performance	–0.060	−0.099*	−0.137**	0.112*	0.298**	0.352**	0.402**	1	
9. Extra-role performance	−0.120*	−0.139**	–0.066	0.120*	0.221**	−0.174**	0.200**	0.309**	1
Mean	1.46	2.86	2.30	25.13	3.72	3.38	–0.01	3.17	3.69
SD	0.499	0.885	0.792	29.207	0.734	0.949	1.331	0.996	0.982

N = 403; *p < 0.05, and **p < 0.01.

### Hypothesis Testing

The results of hierarchical regression analysis are reported in Table 3. To test the direct effect of supervisor positive feedback on subordinate in-role performance and extra- role performance, we conducted hierarchical regression analyses controlling for the gender, age, education and tenure of subordinates. Model 2 shows that supervisor positive feedback is positively related to subordinate in-role performance (β = 0.287, *p* < 0.001). Model 7 shows that supervisor positive feedback is also positively related to subordinate extra-role performance (β = 0.201, *p* < 0.001). Therefore, Hypothesis 1a and Hypothesis 1b are supported.

**TABLE 3:** Hierarchical regressions for main study variables.

Variables	Subordinate in-role performance					Subordinate extra-role performance				
	Model 1	Model 2	Model 3	Mode 4	Model 5	Model 6	Model 7	Model 8	Model 9	Model 10
Gender	–0.032	–0.024	–0.026	–0.041	–0.041	–0.097	–0.092	–0.089	–0.093	–0.093
Age	–0.052	–0.013	–0.026	–0.027	–0.043	−0.112*	–0.0085	–0.089	−0.126*	−0.134**
Education	−0.166**	−0.157**	−0.148**	−0.160**	−0.145**	–0.067	–0.068	–0.046	–0.069	–0.061
Tenure	0.157**	0.151**	0.122**	0.158**	0.131**	0.131*	0.127*	0.107*	0.131**	0.114*
RF			0.367***		0.342***			0.185***		−0.231***
SPF		0.287***	0.216***				0.201***	0.134**		
SPF × RF			0.178***					0.118*		
SNF				0.347***	0.284***				−0.183***	−0.224***
SNF × RF					–0.060					–0.014
*R* ^2^	0.047	0.128	0.257	0.167	0.285	0.047	0.087	0.130	0.081	0.133
Δ*R*^2^		0.081	0.129	0.120	0.118		0.049	0.043	0.034	0.052
*F*	4.947*	11.681***	19.517***	15.908***	22.450***	4.948***	7.566***	8.426***	6.977***	8.643***

*N = 403.*

SPF represents supervisor positive feedback; SNF represents supervisor negative feedback; RF represents regulatory focus; ***p < 0.001, **p < 0.01, *p < 0.05.

Similarly, for the direct effect of supervisor negative feedback on subordinate in-role performance and extra- role performance, the results of hierarchical regression analyses indicate supervisor negative feedback is positively related to subordinate in-role performance (Model 4: β = 0.347, *p* < 0.001) and negatively related to subordinate extra-role performance (Model 9: β = −0.183, *p* < 0.001). Hence, Hypothesis 2a and Hypothesis 2b are supported.

**FIGURE 2:**
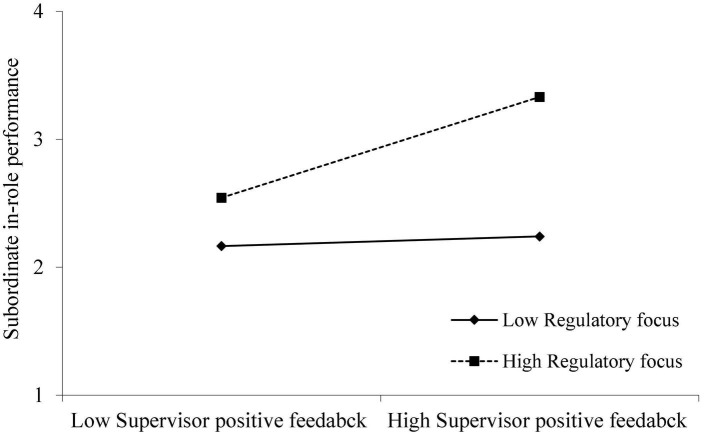
Interaction of supervisor positive feedback and regulatory focus on subordinate in-role performance.

To test the moderating role of regulatory focus, we followed Cohen et al. (2003), Preacher et al. (2010) and Hayes’s (2015) procedures. For its moderating effect in the relationship between supervisor positive feedback and subordinate in-role performance, Model 3 shows the interaction term of supervisor positive feedback and regulatory focus is positively and significantly related to subordinate in-role performance (β = 0.178, *p* < 0.001). Then, we applied parametric bootstrap to estimate the CI around the indirect effect of regulatory focus (Preacher et al., 2010). The results indicate this indirect effect is stronger when subordinates have high regulatory focus [Estimate = 0.3729, 95% CI = (0.2006, 0.5453)], and weaker when subordinates have low regulatory focus [Estimate = 0.1960, 95% CI = (0.0396, 0.3425)]. In addition, we plotted this interaction as a conditional value of regulatory focus (one standard deviation above and below the mean). As displayed in Figure 2, supervisor positive feedback more positively related to subordinate in-role performance when the subordinates have promotion focus. Hence, Hypothesis 3a was supported.

**FIGURE 3:**
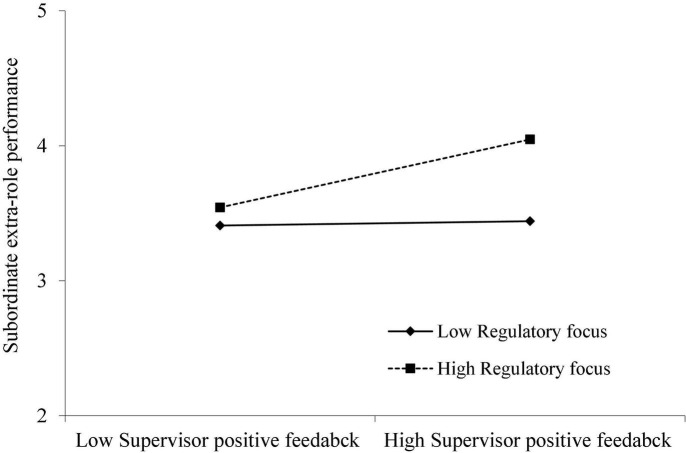
Interaction of supervisor negative feedback and regulatory focus on subordinate extra-role performance.

For the moderating effect of regulatory focus in the relationship between supervisor positive feedback and subordinate extra-role performance, Model 8 shows the interaction term of supervisor positive feedback and regulatory focus is also positively and significantly related to subordinate extra-role performance (β = 0.118, *p* < 0.05). The results of bootstrap analysis show this indirect effect is also stronger when subordinates with high regulatory focus [Estimate = 0.3981, 95% CI = (0.2150, 0.5812)], and weaker when subordinates with low regulatory focus [Estimate = 0.2671, 95% CI = (0.1391, 0.3951)]. The pattern of the interaction effect is displayed in Figure 3. Thus, Hypothesis 3b was supported.

For the moderating effect of regulatory focus in the relationship between supervisor negative feedback and subordinate in-role performance, Model 5 shows the interaction term of supervisor negative feedback and regulatory focus isn’t significantly related to subordinate in-role performance (β = −0.060, *p* > 0.05). And, Model 10 shows the interaction term of supervisor negative feedback and regulatory focus is also not significantly related to subordinate extra-role performance (β = −0.014, *p* > 0.05). These two results indicate that the moderating effect regulatory focus in the relationship between supervisor negative feedback subordinate in-role and extra-role performance are not significant. Therefore, Hypothesis 3c and Hypothesis 3d are not supported.

## Discussion

This study has built and verified a theoretical model to explore the differences of supervisor positive feedback and negative feedback on subordinate in-role performance and extra-role performance, and the moderating role of regulatory focus in these relationships. With data from pairing samples of 403 Chinese employees and their direct supervisors, the results revel that supervisor positive feedback is positively related to subordinate in-role and extra-role performance. Supervisor negative feedback is positively related to subordinate in-role performance and negatively related to subordinate extra-role performance. Regulatory focus of subordinate can moderate the influence of supervisor positive feedback on subordinate in-role and extra-role performance, but it cannot moderate the influence of supervisor negative feedback on subordinate in-role and extra-role performance. That means when subordinates have promotion focus, the influence of supervisor positive feedback on their in-role performance and extra-role performance was stronger than those with prevention focus.

### Theoretical Implications

The findings of this study contribute to the literatures on supervisor feedback and subordinate performance in several ways. Firstly, this study explores the different effects of positive and negative feedback from supervisors on subordinate in-role and extra-role performance. Existing studies have confirmed that both positive and negative feedback from supervisors have an important impact on subordinate performance (Kluger and DeNisi, 1996). However, there is no consensus among scholars on whether this effect is positive or negative (Podsakoff and Farh, 1989; Trope and Neter, 1994; Dahling et al., 2017). Therefore, this study further divides subordinate performance into two types: in-role performance and extra-role performance, so as to help us more accurately grasp the different impacts of supervisor positive and negative feedback on subordinate performance. The empirical analyses of this study revel that supervisor positive feedback has a positive impact on subordinate in-role performance and extra-role performance, and supervisor negative feedback has a positive impact on subordinate in-role performance, but a negative impact on subordinate extra-role performance. This study is one of the first to explore the different effects of supervisor positive feedback and negative feedback on subordinate in-role performance and extra-role performance.

Secondly, the feedback mental process model points out that the feedback receivers’ responses to feedback is not only affected by external environmental factors, but also affected by their own individual characteristics (Ilgen et al., 1979). For the influence of supervisor feedback on subordinates, individual characteristics of subordinates play a significant moderating role (Jaworski and Kohli, 1991; Su et al., 2019). Hence, this study introduces regulatory focus as a moderator of the relationship between supervisor feedback and subordinate performance, and verifies that individuals with different regulatory focus have different behaviors when facing supervisor feedback in daily work (Lockwood et al., 2002; Li C. et al., 2020). These findings further deify the extension of the influence of supervisor feedback on subordinates from the perspective of individual differences of subordinates, and clearly demonstrate the external boundary conditions of the influence of supervisor positive and negative feedback on subordinate in-role and extra-role performance.

Lastly, the conclusions of this study extend the breadth and depth of the Regulatory Focus Theory. Based on Chinese context, this study confirms that regulatory focus of subordinates positively moderates the influence of supervisor positive feedback on subordinate in-role performance and extra-role performance. This supports the results conducted by Higgins (2000), which highlight that individual regulatory focus can moderate the influence of situational factors such as leadership on individual attitude, behavior and performance. And for all we know, this study is the first to introduce as regulatory focus a moderator into the exploration of the relationship between supervisor feedback and subordinate performance. This also echoes the calls of many previous scholars (e.g., Higgins, 1997, 2005; Lockwood et al., 2002; Vaughn, 2017; Li C. et al., 2020) to explore the role of regulatory focus in the fields of Management Psychology and Organizational Behavior.

### Practical Implications

As an important behavior modification tool and incentive strategy in organizations, feedback is widespread and plays a significant role in the daily operation of organizations (London, 1995; Su et al., 2019). However, how to give feedback effectively has always been a difficult problem faced by the organization managers, especially the front-line managers. In view of this, this study verifies the different effects of supervisor positive and negative feedback on subordinate in-role and extra-role performance. These conclusions have the following two implications for specific organizational management practice.

On the one hand, supervisor feedback is an important factor affecting subordinate performance. Supervisor positive feedback has a positive effect on subordinate in-role and extra-role performance, while negative feedback has a positive impact on subordinate in-role performance, and a negative impact on subordinate extra-role performance. Because of this, the organization and its managers should pay full attention to the organizational feedback and realize the importance of supervisor feedback. In daily work, managers should not only actively convey information to subordinates through feedback to help them complete their work better, but also timely respond to the feedback wishes of subordinates and encourage them to actively express their opinions. In addition, when giving daily feedback to subordinates, supervisors should timely give guidance and feedback in a targeted and personalized manner according to the actual situation of subordinates. Only in this way can the supervisors help the subordinates to continuously improve their own ability and make full use of the positive role of supervisor feedback.

On the other hand, for the moderating role of regulatory focus, managers are suggested take individual personality traits into account and provide personalized feedback to their subordinates. Supervisor feedback is an art, and for subordinates with prevention focus, their response to supervisor positive feedback is more obvious. Therefore, in the daily management practice, managers should not only actively give positive feedback to subordinates, but also observe and analyze carefully, and give differentiated feedback to subordinates with different regulatory focus, especially those with promotion regulatory focus. Besides, in the selection process of the organization, it is necessary to consider, hire and promote candidates who have promotion regulatory focus. This can also fundamentally improve the likelihood that members of the organization will respond positively to feedback from their superiors.

### Limitations and Recommendation

Despite the above contributions, this study inevitably has some limitations. The first is the representativeness of the study sample. Although we collected the data from two sources (supervisors and subordinates) at two different time to reduce common method biases (Podsakoff et al., 2012), the measurements of supervisor positive feedback, negative feedback, promotion and prevention regulatory focus were still measured by using subordinate’s self-perception at the same time. Hence, an experimental design is suggested to discuss the possible dynamic relationships between these variables. Meanwhile, we only collected samples from Chinese companies, which may limit the generalization of our conclusions to specific cultural profiles. Numerous previous studies have verified that cultural background may be an important factor affecting subordinate’s interpretation of supervisor feedback (e.g., London, 1995; Peng and Chiu, 2010; Zheng et al., 2015; Su et al., 2019). We therefore encourage future research to replicate the current research in other specific cultures, particularly in the Western context. In addition, this study just introduced regulatory focus as moderator in the relationship between supervisor feedback and subordinate performance, there are probably other moderators that can affect this relationship. For example, we suggest that future studies use feedback orientation (London and Smither, 2002) and feedback attribution (Hempel, 2008) as moderators to examine boundary conditions in which supervisor feedback affects subordinates.

## Data Availability Statement

The raw data supporting the conclusions of this article will be made available by the authors, without undue reservation.

## Ethics Statement

The studies involving human participants were reviewed and approved by Ethical committee of Capital Normal University. The participants provided written informed consent to participate in this study.

## Author Contributions

WS, SY, and QQ were responsible for and participated in the present study. WS proposed the research idea, designed the research, wrote and revised the manuscript. SY made some contributions in research design and critical revision. QQ took part in research design, data collection and data analysis. All authors contributed to the article and approved the submitted version.

## Conflict of Interest

The authors declare that the research was conducted in the absence of any commercial or financial relationships that could be construed as a potential conflict of interest.

## Publisher’s Note

All claims expressed in this article are solely those of the authors and do not necessarily represent those of their affiliated organizations, or those of the publisher, the editors and the reviewers. Any product that may be evaluated in this article, or claim that may be made by its manufacturer, is not guaranteed or endorsed by the publisher.
